# Millepachine, a potential topoisomerase II inhibitor induces apoptosis via activation of NF-κB pathway in ovarian cancer

**DOI:** 10.18632/oncotarget.10739

**Published:** 2016-07-20

**Authors:** Wenshuang Wu, Buyun Ma, Haoyu Ye, Taijin Wang, Xiaoyan Wang, Jianhong Yang, Yuquan Wei, Jingqiang Zhu, Lijuan Chen

**Affiliations:** ^1^ State Key Laboratory of Biotherapy/Collaborative Innovation Center of Biotherapy and Cancer Center, West China Hospital of Sichuan University, Chengdu, China; ^2^ Department of Thyroid and Breast Surgery, West China Hospital of Sichuan University, Chengdu, China; ^3^ Department of Ultrasound, West China Hospital of Sichuan University, Chengdu, China

**Keywords:** topoisomerase II inhibitor, millepachine, apoptosis, NF-κB pathway, ovarian cancer

## Abstract

Millepachine (MIL) was a novel chalcone that was separated from *Millettia pachycarpa* Benth (Leguminosae). We found MIL induced apoptosis through activating NF-κB pathway both in SK-OV-3 and A2780S cells. Western blot showed that MIL increased the levels of IKKα, p-IKKα/β, p-IκBα and NF-κB (p65) proteins, and decreased the expression of IκBα protein. Immunohistochemistry analysis indicated that translocation of NF-κB into the nucleus increased in both ovarian cancer cells. EMSA assay proved MIL enhanced NF-κB DNA-binding activity in the nuclear. That specific NF-κB inhibitors alleviated MIL-induced apoptosis suggested NF-κB activation showed a pro-apoptotic function in SK-OV-3 and A2780S cells. Since NF-κB could be activated by double strand breaks and showed a pro-apoptotic function in the DNA damage response, SCGE assay and western blot revealed that MIL caused DNA strand breaks and significantly increased the level of p-ATM protein and further increased the levels of p-IKKα/β and NF-κB (p65) protein in SK-OV-3 and A2780S cells, while a specific ATM inhibitor could alleviated these effects. Moreover, Topoisomerase II drug screening kit and computer modeling assay were used to prove that MIL induced the production of linear DNA and inhibited the activity of topoisomerase II through binding with Topoisomerase II-Cleaved DNA complex to stabilize the complex. Taken together, our results identified that MIL exhibited anti-tumor activity through inhibiting topoisomerase II activity to induce tumor cells DNA damage, and MIL-activated NF-κB pathway showed a pro-apoptotic function in response to DNA damage.

## INTRODUCTION

Genomic stability is constantly threatened by DNA damage arising from numerous intrinsic and environmental sources [1, 2]. Failure to repair DNA damage can lead to mutations, genomic instability, premature aging, mental multiplex retardation and other developmental disorders as well as cancer. Consequently, cells have involved a sophisticated signal of DNA repair mechanisms to sense different types of DNA damage and safeguard the genome, which was called DNA damage response (DDR) [3, 4]. The DDR would activate DNA repair and DNA damage signaling pathways by phosphorylating the downstream proteins including ataxia-telangiectasia mutated (ATM), ATM and Rad3-related (ATR) and DNA-dependent protein kinases (DNA-PK) [5–7]. Depending on the cell type and the severity and extent of DNA damage, the activated DDR can elicit different cellular responses. Mild DNA damage normally leads to the induction of cell cycle arrest, whereas severe and irreparable injury leads to the cellular response towards induction of senescence or cell death programs, such as apoptosis, mitotic catastrophe, autophagy and other processes [1, 8].

Induction of DNA damage is the mechanism of most anti-cancer drugs [9]. These drugs include cispaltin and carboplatin causing cross-linking of DNA and ultimately triggering apoptosis, alkylating agent temozolomide, topoisomerase inhibitors such as irinotecan and etoposide, and PARP inhibitors such as Olaparib and BSI-201, which demonstrate great potential in various cancer treatments in clinical trials [10–12]. Furthermore, drugs inducing DNA damage are more likely to target highly proliferative cancer cells.

DNA topoisomerases are enzymes that regulate the overwinding or underwinding of DNA, which are divided into two classes: topoisomerase I cuts and reanneals one strand of a DNA double helix to induce DNA single-strand break (SSB), and topoisomerase II cuts and reanneals both strand of one DNA double helix to induce DNA double-strand break (DSB) [13]. Topoisomerase II is the target of important chemotherapeutic agents. Most topoisomerase II inhibitors have been in clinical use for many years, such as etoposide, doxorubicin and mitoxantrone [14–16].

Millepachine (MIL), a novel natural chalcone separated from *Millettia pachycarpa* Benth, has been reported in our previous study to induces G2/M arrest by inhibiting CDK1 activity and causing apoptosis via ROS-mitochondrial apoptotic pathway in human hepatocarcinoma cells *in vitro* and *in vivo* [17]. Here, we found that MIL showed significant anti-tumor activity against human ovarian cancer *in vitro* and *in vivo*. Further study revealed that MIL exhibited anti-tumor activity through inhibiting topoisomerase II activity to induce tumor cells DNA damage, and MIL-activated NF-κB pathway showed a pro-apoptotic function in response to DNA damage.

## RESULTS

### MIL strongly inhibited proliferation of human ovarian cancer *in vitro* and *in vivo*

**Figure 1:**
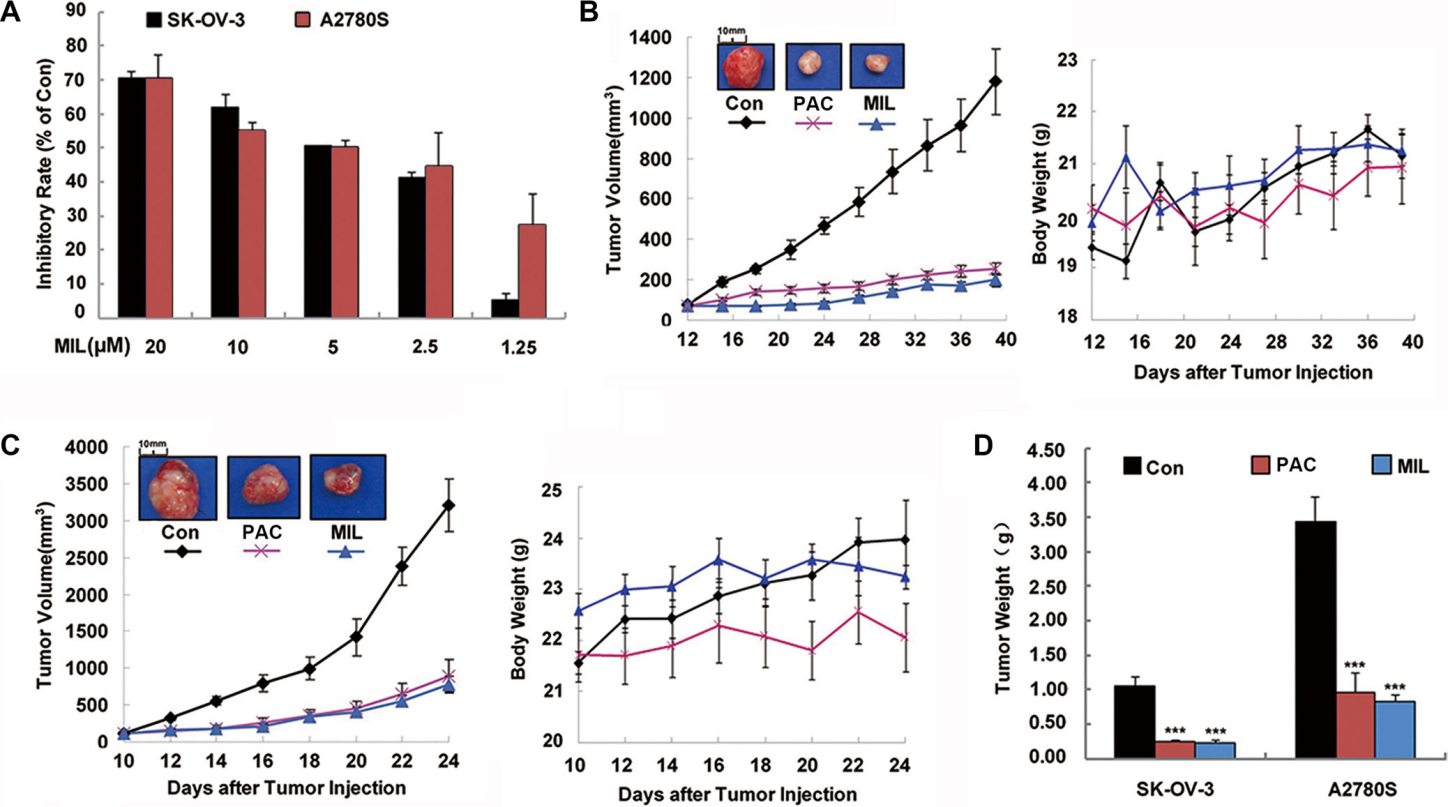
MIL strongly inhibited proliferation of human ovarian cancer *in vitro* and *in vivo* (**A**) The inhibitory effect of MIL on cell proliferation. The inhibition of cell proliferation was determined using MTT assay. The cells were treated with the indicated concentrations of MIL for 48 h. The experiment was repeated at least three times. (**B**) and (**C**) Tumor volume-time and body weight-time curve in SK-OV-3 and A2780S xenograft model. Treatment was initiated when the average size of the tumor reached 100 mm^3^. MIL-treated group were treated with indicated dosage in 200 μL of physiological saline of MIL every two days at a dosage of 20 mg/kg. The positive group received PAC at a dosage of 5 mg/kg every two days, and the control group received injection of physiological saline alone as test groups. (**D**) The bar charts of tumor weight. *Columns*, means; bars, SD (*n* = 6). ***P < 0.001, significantly different compared with control by *t*-test.

Ovarian cancer is the most common cause of cancer death from gynecologic tumors [18]. It is warranted to develop and test new therapeutic agents due to the high mortality rate. In this research, two ovarian cancer cell lines, SK-OV-3 and A2780S cells, were chosen to investigate the anti-tumor activity of MIL in ovarian cancer cells. MTT assay showed that MIL dose-dependently inhibited the proliferation of both SK-OV-3 and A2780S with IC_50_ values of 4 μM after 48 h treatment (Figure 1A).

To further verify the inhibitory effect of MIL on tumor growth *in vivo*, we examined the anti-tumor effect of MIL on the growth of ovarian cancer cells in nude mice. MIL was administered *i.v.* at 20 mg/kg every two days in a volume of 200 μL, made up in 0.9% saline with a few drops Tween 80 (2.5%) and ethanol (2.5%). At the end of the experiment, MIL treatment group significantly inhibited the tumor growth in both SK-OV-3 (Figure 1B and 1D) and A2780S xenograft models (Figure 1C, 1D) with the inhibitory rate of 80.31% and 75.79%, respectively (Supplementary Table S1). The average tumor weights of MIL-treated groups were 0.22 ± 0.04 g (*P* < 0.001) for SK-OV-3 and 0.83 ± 0.10 g (*P* < 0.001) for A2780S tumor models in comparison to 1.13 ± 0.15 g and 3.44 ± 0.35 g of control groups (Figure 1D). Notably, MIL at 20 mg/kg *i.v* administration exhibited better tumor inhibition than positive drug (paclitaxel (PAC)) at 5 mg/kg (Supplementary Table S1). In addition, no body weight loss was observed during the treatment of MIL in two xenograft models (Figure 1B, 1C).

### MIL induced apoptosis in ovarian cancer cells

**Figure 2:**
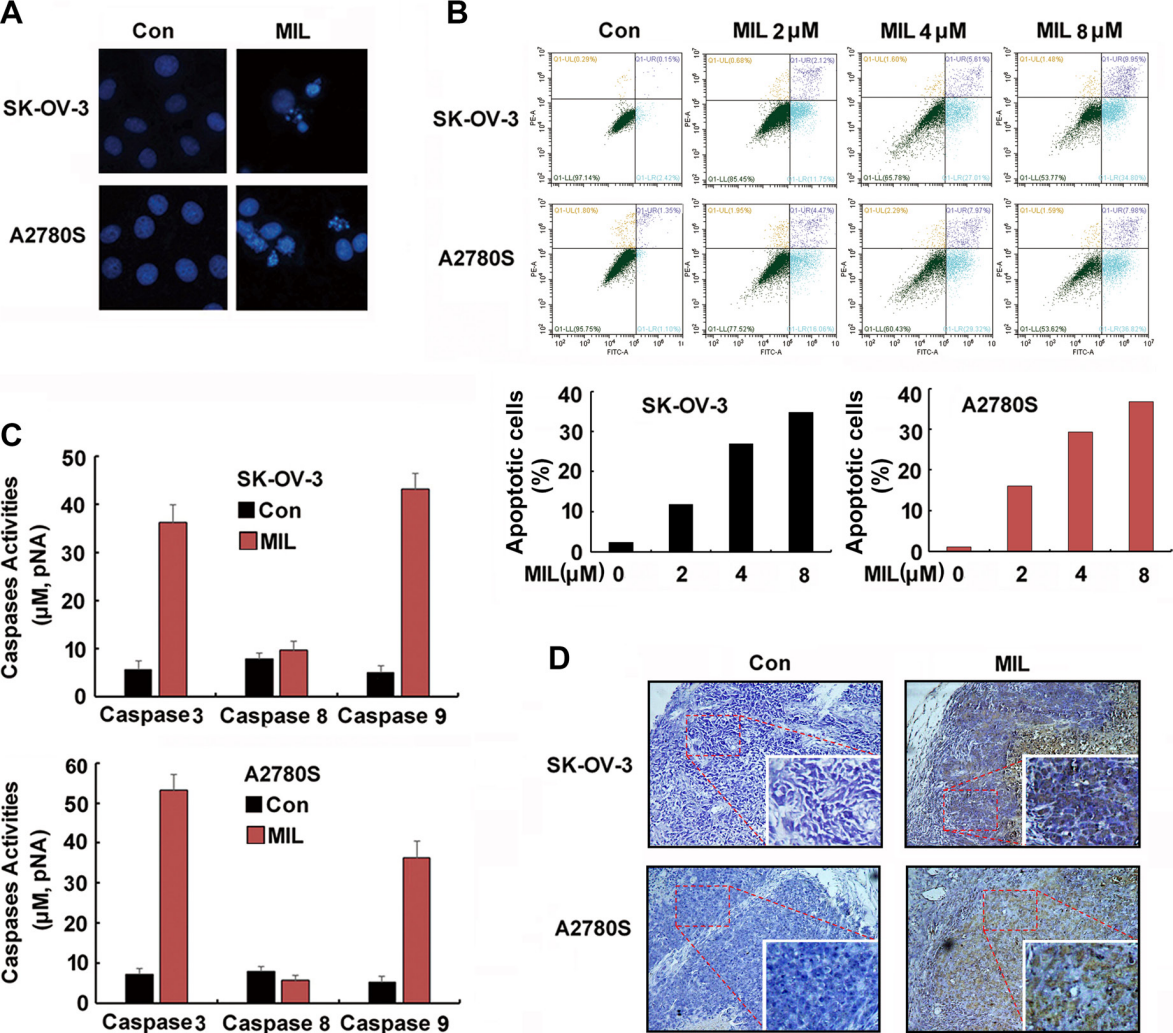
MIL induced apoptosis in both human ovarian cancer cells (**A**) The changes of cell nuclei (200×). The nuclei of SK-OV-3 and A2780S cells were stained by Hoechst 33258 after treated with indicated 4 μM MIL for 48 h. The changes of nuclei of cells were observed by using an inverted fluorescence microscopy. The experiment was repeated at least three times with similar results. (**B**) MIL induced apoptosis of human ovarian cancer cells. SK-OV-3 and A2780S cells were treated with different concentration MIL for 48 h. Cells were stained with Annexin V-FITC and PI for 15 min in dark, then analyzed on a flow cytometer. The number in the right quads of each panel means the percentage of Aannex in V positive cells. The experiment was repeated three times yielding similar results. (**C**) MIL activated caspase 3 and 9 in SK-OV-3 and A2780S cells. Cells were treated with 4 μM MIL for 36 h. Cells were then lysed and caspases activity was measured using a colorimetric test system. The experiment was repeated three times. (**D**) TUNEL assay of tumor. The apoptosis of tumor were determined using a TUNEL kit.

To further investigate the antitumor mechanism of MIL, we first observed the morphology of SK-OV-3 and A2780S cells after treatment of MIL. We found obvious change of cells with characteristics of cell shrinking, nucleus breaking into apoptotic bodies with MIL treatment for 48 h (Figure 2A). The results of flow cytometry with propidium iodide (PI) and annexin V-FITC labeling proved that MIL induced concentration-dependent apoptosis in SK-OV-3 with the percentage of apoptosis cells from 11.75%–34.8% compared with 2.42% for the control group. MIL also induced apoptosis in A2780S cells in concentration-dependent manner (Figure 2B). As caspases play key roles in programmed cell apoptosis, the activity of caspases in two ovarian cancer cells was also measured with a colorimetric test system. As shown in Figure 2C, the activity of caspase 3 and 9 had an obvious increase, but the activity of caspase 8 had little changes between control and MIL-treatment cells in both ovarian cancer cells. Furthermore, immunohistochemical of TUNEL confirmed that MIL displayed a strong apoptosis inducing effect in MIL-treated tumor tissues (Figure 2D).

### MIL activated NF-κB and NF-κB-related pathway in ovarian cancer cells

**Figure 3:**
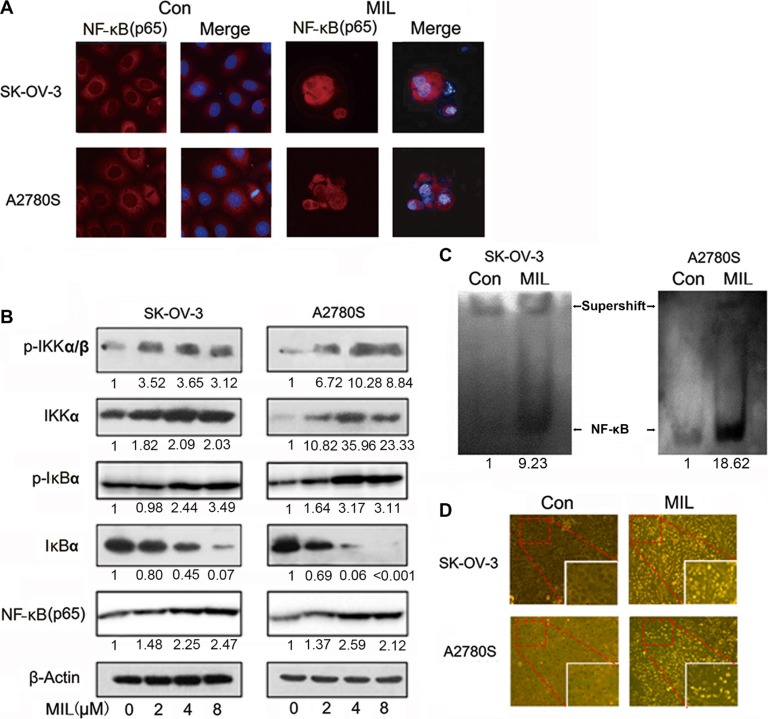
MIL activated NF-κB and NF-κB-related pathway in ovarian cancer cells (**A**) Immunofluorescence of NF-κB (200×). SK-OV-3 and A2780S cells were treated with 4 μM MIL for 48 h. NF-κB(p65) was detected by a fluorescence microscopy as described in Material and Methods. Data from a typical experiment were shown, repeated three times with similar results. The experiment was repeated three times with similar results. (**B**) NF-κB activity related protein analysis. SK-OV-3 and A2780S cells were treated with varying concentrations of MIL for 48 h. The protein levels of IKKα, p-IKKα/β, IκBα, p-IκBα and NF-κB (p65) were determined using western blot assay. The experiment was repeated at least three times. (**C**) MIL promoted NF-κB binding with DNA in SK-OV-3 and A2780S cells. The experiment was repeated three times. (**D**) MIL activated NF-κB pathway in the tumors of SK-OV-3 and A2780S xenograft models. The expressions of NF-κB (p65) in tumors were detected using immunohistochemistry (100×).

Since NF-κB played an important role in cell apoptosis [19], we investigated the effect of MIL on NF-κB pathway. Interestingly, we found that MIL activated NF-κB pathway in SK-OV-3 and A2780S cells. MIL did not only increase the expression of NF-κB (p65) in cytoplasm but also enhanced the expression of NF-κB (p65) in the nucleus of both ovarian cancer cells (Figure 3A). Western blot further showed that MIL also increased the levels of IKKα, p-IKKα/β, p-IκBα and NF-κB (p65) protein, and deceased the expression of IκBα protein (Figure 3B). To investigate the translocation of NF-κB into the nucleus of both ovarian cancer cells, EMSA assay was used to determine the effect of MIL on the activity of NF-κB DNA-binding. As depicted in Figure 3C, MIL indeed enhanced NF-κB DNA-binding activity in both ovarian cancer cells. These data revealed that MIL significantly activated NF-κB in SK-OV-3 and A2780S cells. In consistent to *in vitro* results, immunohistochemistry analysis of NF-κB (p65) showed that MIL increased the expression of NF-κB (p65) protein in MIL-treated tumor tissue (Figure 3D).

### Activated-NF-κB showed a pro-apoptotic function in two ovarian cancer cells

**Figure 4:**
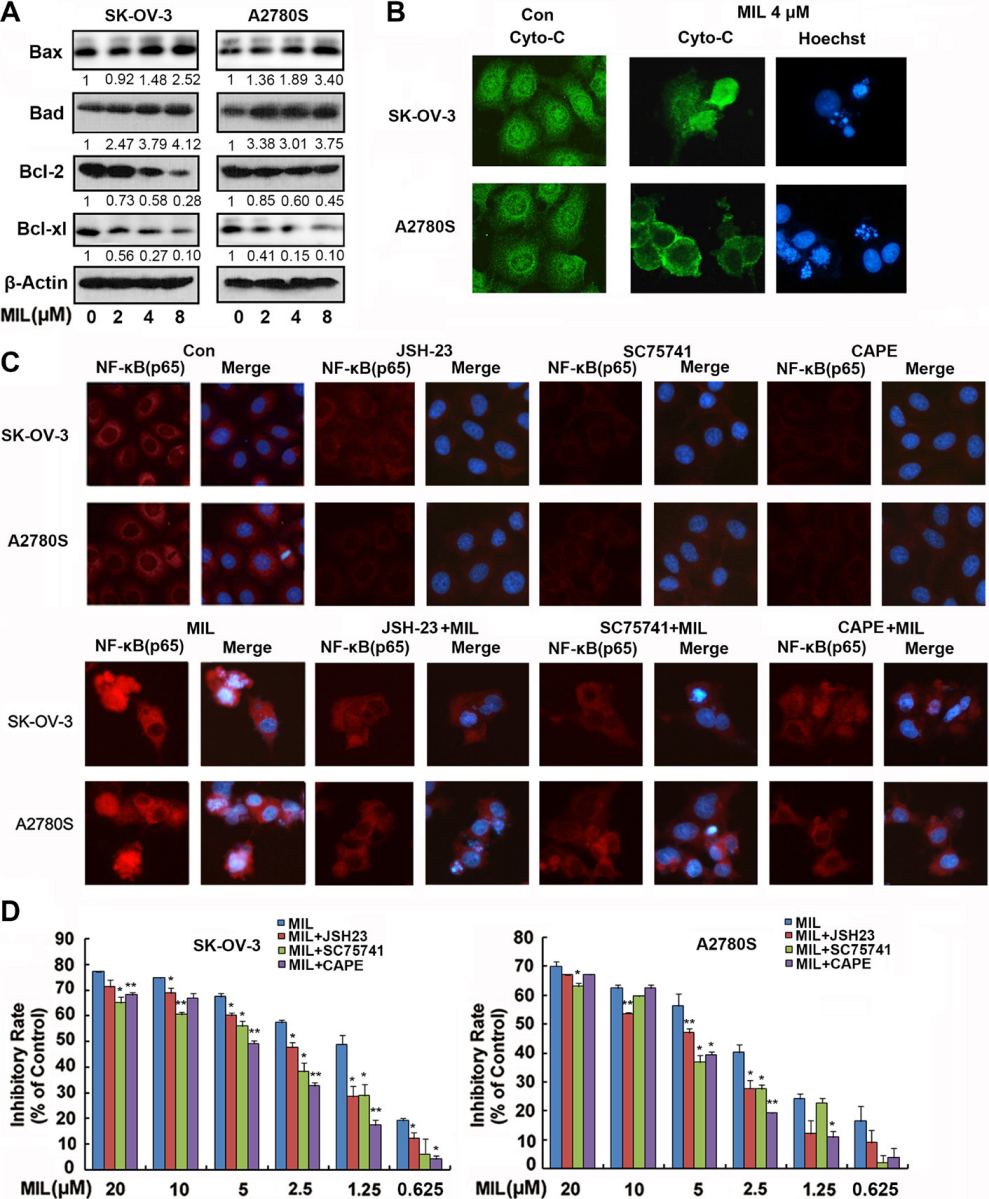
Activated-NF-κB showed a pro-apoptotic function in MIL treated apoptotic ovarian cancer cells (**A**) MIL increased the level of Bax, Bad and decreased the expression of Bcl-2 and Bcl-xl proteins in SK-OV-3 and A2780S cells. (**B**) Immunofluorescence of cytochrome c. Cytochrome c was detected by a fluorescence microscopy (200×). The nuclei of cells were stained by Hoechst 33258. (**C**) Immunofluorescence of NF-κB (p65) (200×). SK-OV-3 and A2780S cells were treated with different compounds (vehicle, 5 μM JSH-23, 5 μM SC75741, 5 μM CAPE, 4 μM MIL, 4 μM MIL combined with pre-treated 5 μM JSH-23, 4 μM MIL combined with pre-treated 5 μM SC75741, 4 μM MIL combined with pre-treated 5 μM CAPE) for 48 h. NF-κB(p65) was detected by a fluorescence microscopy. The nuclei were stained with Hoechst 33258. (**D**) The inhibitory effect of MIL on cell proliferation in the presence of specific NF-κB inhibitors JSH-23, SC75741 and CAPE. *Columns*, means; bars, SD (*n* = 3). *P < 0.05, **P < 0.01, significantly different compared with control by *t*-test. All experiments were repeated at least three times with nearly identical results.

Normally, NF-κB regulates apoptosis through regulating the expression of target genes, such as increasing the expression of Bcl-2 and inhibiting cytochrome C releasing from mitochondria to inhibit apoptosis [20]. However, our results revealed that MIL decreased the expression of Bcl-2 and Bcl-xl and increased the level of Bax (Figure 4A), enhanced the release of cytochrome C from mitochondria into cytoplasm in both MIL-induced apoptotic cells (Figure 4B), exhibiting obvious characteristics of apoptosis. To further clarify whether NF-κB contributes to MIL-induced apoptosis, specific NF-κB inhibitors (JSH23, SC75741 and Caffeic Acid Phenethyl Ester (CAPE)) were used to investigate MIL's effect on SK-OV-3 and A2780S cells by by immunofluorescence and MTT [21–23]. JSH23, SC75741 and CAPE at 5 μM concentration inhibited the expression of NF-κB (p65) in both SK-OV-3 and A2780S cells. MIL significantly increased the expression of NF-κB (p65) in cytoplasm and nucleus and MIL combined with NF-κB inhibitors did not only decrease the effect to up-regulate the expression of NF-κB of MIL (Figure 4C), but also reduced the effect to inhibit tumor cells proliferation of MIL. MTT results showed the NF-κB signaling blockade could alleviate the anti-proliferative effect of MIL on two ovarian cancer cells (Figure 4D). These data suggested that activation of NF-κB showed a pro-apoptotic function in MIL-treated SK-OV-3 and A2780S cells.

### MIL induced DNA damage in ovarian cancer cells

**Figure 5:**
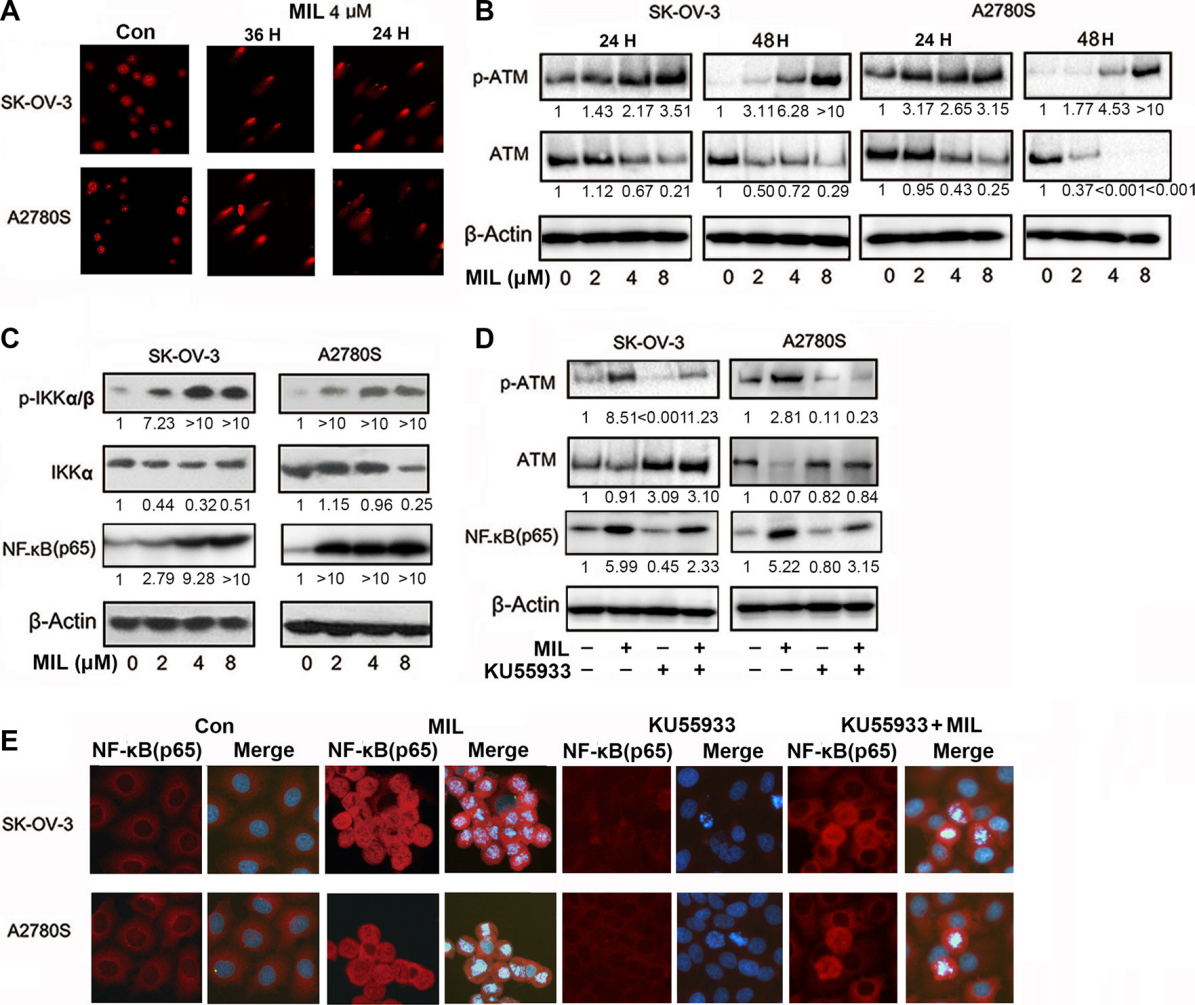
MIL induced DNA damage in ovarian cancer cells (**A**) Comet assay was performed in SK-OV-3 and A2780S cells after 24 h or 36 h of MIL treatment, tail moments were measured and photographs were captured at 200×. (**B**) MIL increased the expression of p-ATM proteins in SK-OV-3 and A2780S cells. (**C**) MIL increased the expression of NF-κB-related protein within 24 h treatment. (**D**) KU55933 inhibited the levels of p-ATM and NF-κB (p65) protein. (**E**) Immunofluorescence assay (200×) exhibited that KU55933 inhibited the increasing effect of MIL on the expression of NF-κB with 24 h treatment in SK-OV-3 and A2780S cells. All experiments were repeated at least three times with nearly identical results.

Previous studies reported that NF-κB could be activated by double strand breaks and showed a pro-apoptotic function in the DNA damage response [24, 25]. Our previous study indicated that MIL induced DNA damage in human hepatocarcinoma cancer cells [17]. To measure whether MIL induced DNA damage in human ovarian cancer cells, we analyzed the DNA damage in both MIL-treated ovarian cancer cells using SCGE assay. Figure 5A showed MIL (4 μM) caused DNA strand breaks in both SK-OV-3 and A2780S cells within 24 and 36 h treatment (90% of cells showed the comet image, *P* < 0.01). Mild DNA damage normally leads to cell cycle arrest. ATM, as the main down-regulator of DNA damage could arrest the cell cycle by phosphorylation and activation of cell cycle checkpoint [26]. Since extreme DNA damage is hard to repair, the DNA damage insult is transmitted by the cellular stress response to activation of effectors systems to mediate cell death [27]. After treatment of MIL for 24 and 48 h, MIL induced cells G2/M arrest (Supplementary Figure S1) and apoptosis (Figure 2), respectively and MIL significantly increased the level of p-ATM protein in SK-OV-3 and A2780S cells (Figure 5B). Phosphorylated ATM could further phosphorylate IKK to activate NF-κB following DNA damage [28]. Western blot showed that MIL increased the levels p-IKKα/β and NF-κB (p65) protein with MIL-treatment for 24 h in ovarian cancer cells (Figure 5C). We further investigated the effect of ATM on the activation of NF-κB in MIL-treated SK-OV-3 and A2780S cells in the presence of specific ATM inhibitor (KU55933) [29]. KU55933 could neutralize the effect of MIL to both p-ATM and NF-κB (p65) in some extent in SK-OV-3 and A2780S cells (Figure 5D). The results of immunofluorescence assay proved that KU55933 significantly reduced the expression of NF-κB (p65) in MIL-induced cells (Figure 5E) and MTT assay showed that KU55933 could alleviate MIL-induced apoptosis (Supplementary Figure S2). Thus, above results suggested that the activation of NF-κB was in response to DNA damage and it played an important part in MIL-induced cell apoptosis in human ovarian cells [25, 30].

### MIL inhibited topoisomerase II to induce cell DNA damage

**Figure 6:**
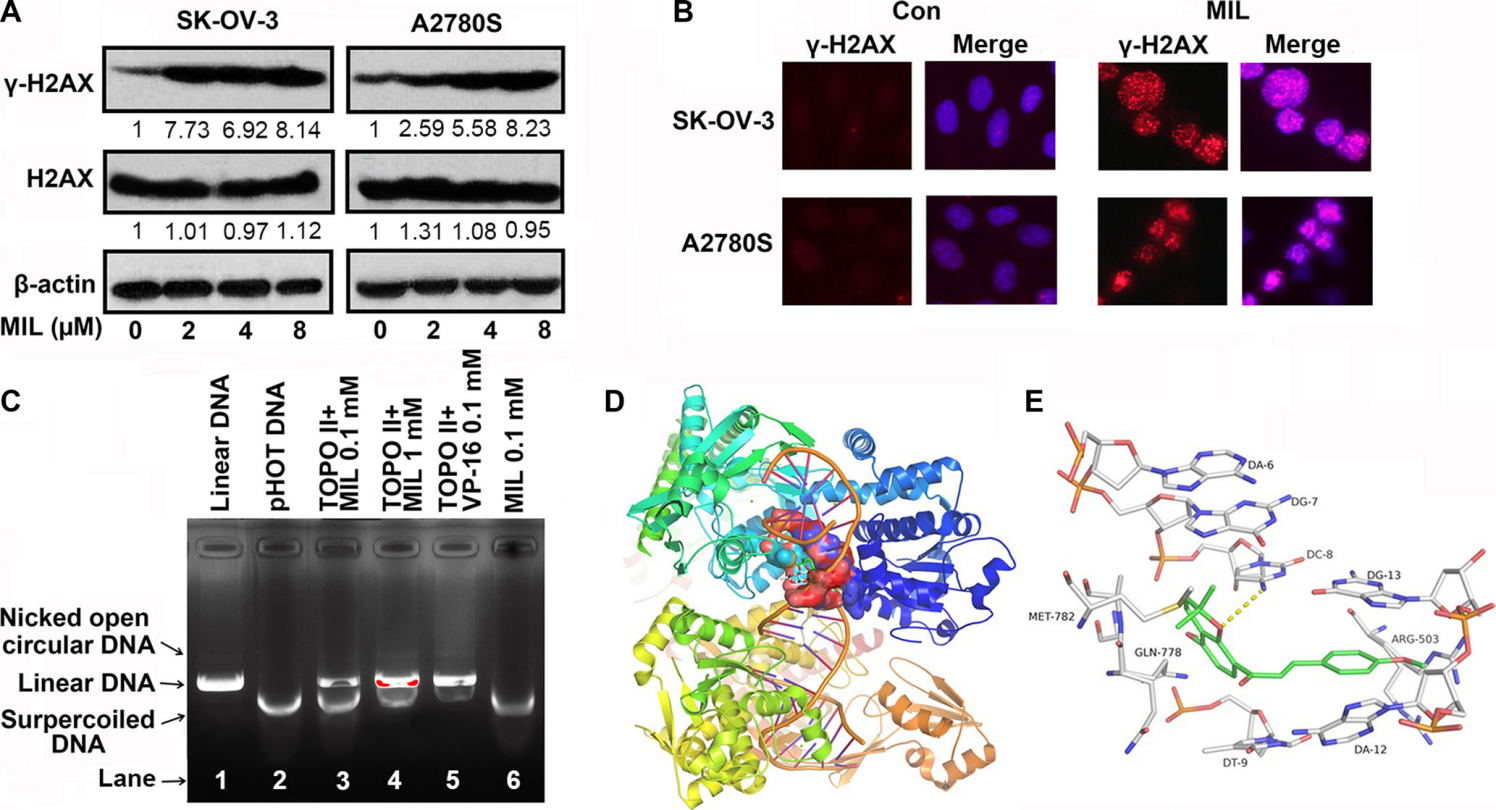
MIL inhibited topoisomerase II to induce cell DNA damage (**A**) MIL increased the level of γ-H2AX in SK-OV-3 and A2780S cells. The experiment was repeated at least three times. (**B**) Immunofluorescence of γ-H2AX (200×). The nuclei of cells were stained by Hoechst 33258. The experiment was repeated three times yielding similar results. (**C**) Action of MIL on topoisomerase II. Supercoiled plasmid DNA (pHOT DNA) was incubated with topoisomerase II and various concentration of MIL or etoposide (VP-16). The reaction of products was separated in 1% agarose gel with 0.5 μg/mL ethidium bromide. The experiment was repeated three times. Overview (**D**) and Close view (**E**) of the potential binding modes of MIL with topoisomerase II beta and DNA in 3HKD.

Normally, DNA damage is divided into DNA single strand breaks (SSB) through inhibiting topoisomerase I and DNA double strand breaks (DSB) *via* inhibiting topoisomerase II [13]. If cells undergo DSB, DNA damage would phosphorylate H2AX at Ser139 (γ-H2AX) [31]. Thus, γ-H2AX was thought to be a marker of DSB. The results of both western blot and immunofluorescence showed MIL increased the level of γ-H2AX protein (Figure 6A, 6B), suggesting that MIL might induce DSB *via* inhibiting topoisomerase II.

Then, we detected the inhibitory effect of MIL on topoisomerase II using topoisomerase II drug screening kit. As shown in Figure 6C, etoposide (VP-16), a topoisomerase II inhibitor, induced the production of linear DNA and caused DNA damage at 0.1 mM concentration (Figure 6C Lane 5). MIL inhibited the activity of topoisomerase II and induced the production of linear DNA in a concentration-dependent manner (Figure 6C Lane 3 and 4), suggesting that MIL targeted topoisomerase II, which had a similar mechanism to VP-16 (Figure 6C).

Compounds that target topoisomerse II are split into two main classes: topoisomerase poisons, targeting the topoisomerase-DNA complex, and topoisomerase inhibitors, disrupting catalytic turnover. [32, 33]. Then, we investigated how MIL interfered with topoisomerse II using computer modeling. The binding mode suggested that MIL interacted with the DNA cleavage parallel to the axis of DNA base pairing in the same style with the known topo II-DNA inhibitors, stabilized by base-stacking π-π interactions between aromatic rings with both upstream and downstream base pairs. MIL could form π-π interactions with DG-12 and DG-13. Moreover, the oxygen of 2H-benzopyran formed H-bonds with the amine of DC-8.

### MIL inhibited proliferation of drug-resistant ovarian cancer cells *in vitro* and *in vivo*

**Figure 7:**
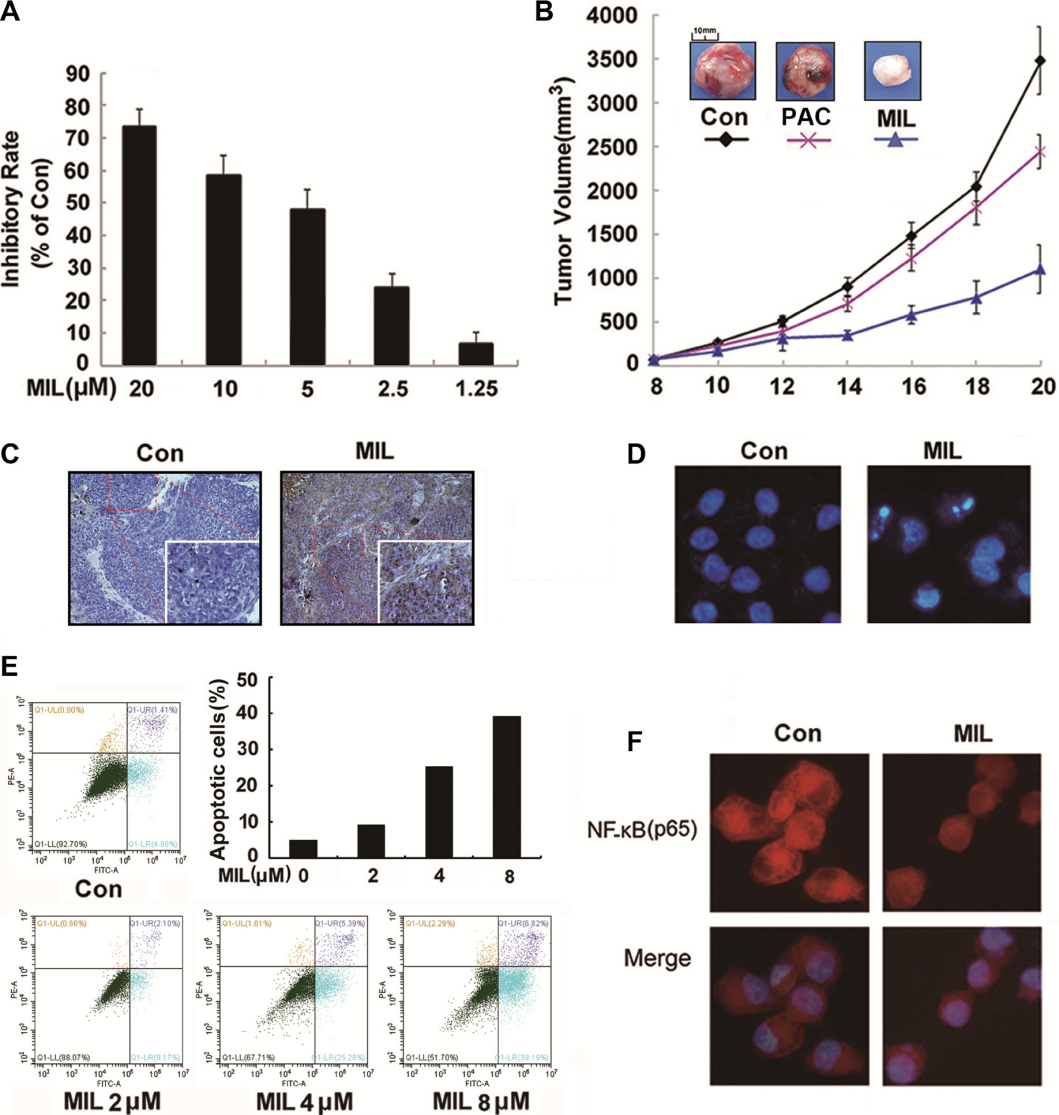
MIL inhibited proliferation in PAC-resistant A2780T cells *in vitro* and *in vivo* (**A**) The inhibitory effect of MIL on the proliferation of PAC-resistant A2780T cells. The cells were treated with the indicated concentrations of MIL for 48 h. The experiment was repeated at least three times. (**B**) Tumor volume-time curve in PAC-resistant A2780T xenograft model. (**C**) MIL induced apoptosis in the tumor of PAC-resistant A2780T xenograft model. The apoptosis of tumor were determined using TUNEL kit. (**D**) The changes of PAC-resistant A2780T cells nuclei (200×). The changes of nuclei of cells were observed by using an inverted fluorescence microscopy. (**E**) MIL induced apoptosis in PAC-resistant A2780T cells using flow cytometry with staining Annexin V-FITC and PI. (**F**) Immunofluorescence of NF-κB in PAC-resistant A2780T cells (200×). The experiment was repeated three times.

As drug resistance is a big problem to successful chemotherapy for ovarian cancer [34], we chose PAC-resistant A2780T cells to investigate inhibition effect of MIL on the proliferation of drug-resistant ovarian *in vitro* and *in vivo*. As depicted in Figure 7A, MIL showed similar proliferation inhibition in PAC-resistant A2780T cells as in A2780S cells, suggesting MIL might show activity in anti-cancer drug resistance. Then we investigated the tumor growth inhibition activity of MIL in the PAC-resistant A2780T cells xenograft model. MIL also showed remarkable reduction of tumor volume and weight in drug-resistant cells in nude mice (Figure 7B, Supplementary Table S1). The average tumor weights of MIL-treated group was 1.46 ± 0.06 g (*P* < 0.001), compared the control group of 3.52 ± 0.30 g and PAC group of 2.51 ± 0.25 g. The inhibitory rate of MIL was 58.48%, higher than PAC of 29.03%.

Meanwhile, MIL also induced apoptosis in MIL-treated tumors (Figure 7C) and in PAC-resistant A2780T cells (Figure 7D, 7E). The results of immunofluorescence assay showed that MIL increased the expression of NF-κB (p65) subunit protein in the nucleus, although NF-κB (p65) protein kept a comparatively high level in cytoplasm of PAC-resistant A2780T cells (Figure 7F).

## DISCUSSION

Ovarian cancer is one of the most deadly gynecologic malignancies worldwide and ranks among top five deadliest cancers in most countries [18, 35]. Although chemotherapeutic agents, such as cisplatin and paclitaxel (PAC), are widely used for the treatment of ovarian cancer, chemo-resistance remains a major therapeutic problem [34, 36]. Hence, it is a critical issue to develop more efficient compounds for improving ovarian cancer chemotherapy treatment. In this paper, MIL showed significant anti-tumor activity in human ovarian cancer, with the inhibitory rate of 80.31% and 75.79% in the SK-OV-3 and A2780S cells xenograft models (Figure 1B, 1C) and 58.48% in PAC-resistant A2780T xenograft model (Figure 7B) (Supplementary Table S1). The significant anti-tumor activity suggested that MIL might be a potential lead compound for cancer drug candidate.

When we studied the underlying mechanism of the anti-tumor activity of MIL in ovarian cancer cells, it was found that MIL activated NF-κB pathway in apoptotic ovarian cancer cells and further research revealed that MIL did not only increase the level of NF-κB (p65) protein (Figure 3A, 3B), but also enhanced NF-κB DNA binding activity (Figure 3C). Specific NF-κB inhibitors (JSH-23, SC75741 and CAPE [21–23]) deceased the expression of NF-κB (p65) (Figure 4C) and alleviated the effect of MIL-induced apoptosis (Figure 4D) implying that NF-κB activation induced by MIL exhibited a pro-apoptotic function in SK-OV-3 and A2780S cells. Several recent studies reported that NF-κB showed a pro-apoptotic function in response to DNA damage induced by chemotherapeutic agents [24, 25]. When cells undergo DNA damage, ATM and ATR were activated in response to DNA damage [37–39], and ATM was primarily activated by auto-phosphorylation on Ser1981 in response to double strand breaks induced by chemotherapeutic drugs [25, 40]. If the DNA damage was too severe to repair, it was transmitted by cellular stress response to mediate cell death [25, 27, 41]. The activated ATM would phosphorylate IKK to activate NF-κB which resulted in a significant increase in DNA damage prior to the induction of apoptosis [42]. The results of SCGE assay revealed that MIL induced significant DNA damage (Figure 5A) and G2/M arrest (Supplementary Figure S1) at 24 h in SK-OV-3 and A2780S cells. MIL increased the level of p-ATM and p-IKK protein in both human ovarian cancer cells. Activated-IKK phosphorylated IκB could separate from NF-κB complex and activated NF-κB. And the results showed that MIL increased the level of activated NF-κB (p65) (Figure 5C). KU55933, an inhibitor of ATM [29], could reduce the effect of MIL to decrease the expression of p-ATM and NF-κB (p65) (Figure 5D). In SK-OV-3 and A2780S cells, MIL caused DNA damage to induce cell cycle arrest and apoptosis through activating ATM. The phosphroylated ATM further activated NF-κB and finally contributed to apoptosis. The results that KU55933 could alleviate MIL-induced apoptosis (Supplementary Figure S2) suggested MIL showed anti-tumor activity through inducing DNA damage.

DNA damage is generally divided into single strand breaks (SSB) and double strand breaks (DSB) [13]. If cells undergo DSB, DNA damage would phosphorylate H2AX at Ser139 (γ-H2AX) to repair DNA [30]. γ-H2AX is used as a biomarker of cellular response to DSB. We proved that MIL increased the expression of γ-H2AX in both SK-OV-3 and A2780S cells using western blot and immmunoflurescence assay (Figure 6A and 6B). Usually, small molecules inducing DNA DSB were through targeting type II topoisomerase [43]. Then, topoisomerase II drug screening kit was used to test the inhibitory activity of MIL on topoisomerase II. MIL induced a significant production of linear DNA revealing MIL might target topoisomerase II to cause DNA damage as VP16 did (Figure 6C). And VP16 could also inhibit the proliferation of SK-OV-3 and A2780S and activated p-ATM and NF-κB (p65) as MIL did (Supplementary Figure S3) Small molecules targeting topoisomerase II are divided into two forms: 1) inhibitors of topoisomerase II, which can inhibit the ATPase activity by acting as noncompetitive inhibitors of ATP, such as HU-331, ICRF-187, ICRF-193, and mitindomide; 2) poisons of topoisomerase II, which can target the DNA-protein complex, such as etoposide, novobiocin, quinolones and teniposide [32, 33, 44]. Results of computer modeling demonstrated that MIL could bind with Topo II-Cleaved DNA complex to stabilize the complex (Figure 6D, 6E). Thus, our data indicated MIL inhibited the activity of topoisomerase II by acting as a topoisomerase II poison.

In conclusion, MIL induced human ovarian cancer cells DNA damage through targeting the topoisomerase-DNA complex. The un-repairable DNA damage caused by the continuing role of MIL induced cell apoptosis to inhibit the tumor growth of human ovarian cancer cells *in vitro* and *in vivo*. And MIL-activated NF-κB showed a pro-apoptotic function in response to DNA damage.

## MATERIALS AND METHODS

### Cell culture and chemicals

The human ovarian cancer cell lines, SK-OV-3 and A2780S, and PAC-resistant A2780T were obtained from American Type Culture Collection (ATCC, Manassas, VA, USA). Cells were cultured in DMEM, supplemented with 10% fetal bovine serum and 50 U/ml penicillin and streptomycin.

MTT, DMSO, Hoechst 33258, PAC and anti-β-actin, Bad and Bcl-2 were purchased from Sigma (St Louis, MO, USA). JSH-23 (S7351), SC75741 (S7273), CAPE (S7414) and KU55933 (S1092) were bought from Selleckchem (USA). Antibodies against Bax, ATM, p-ATM, IKKα, p-IKKα/β, IκBα, p-IκBα, NF-κB (p65), H2AX and γ-H2AX were obtained from Cell Signaling Technology (CST, Danvers, MA). Antibody against Bcl-xl was bought form Abcam (UK). MIL was isolated from *Millettia pachycarpa* Benth. The Sample in all experiments contained MIL of 99% or higher purity, which was dissolved in DMSO to produce a 10 mM stock solution and stored at 4°C. In the *in vivo* experiments, MIL was dissolved in sodium chloride injection containing 2.5% Tween-80 and 2.5% ethanol in a volume of 200 μL.

### Cell proliferation assay

Cells were seeded in 96 well plates and cultured overnight. The cells were treated with different concentration of MIL for 48 h. Cell viability was determined by MTT assay [45]. The absorbance at 570 nm was determined in each well with the Spectramax M5 Microtiter Plate Luminometer (Molecular Devices, USA).

### *In vivo* tumor xenograft

To determine the *in vivo* anti-tumor activity of MIL, cells (5 × 10^6^ in 100 μL saline) were injected s.c. into the right flanks of female nude mice (6 weeks old, BALB/cA-nu (*nu/nu*)). After 2 weeks, the tumors were aseptically dissected and pieces of tumor tissue (2–3 mm^3^ in size) were transplanted s.c. by a trocar into mice. When the tumor size reached 100 mm^3^, mice were randomly divided into groups (*n* = 6) and treated with PAC and MIL every two days. Tumor burden was measured every two days or three days with a caliper (calculated volume (mm^3^) = π/6 × length × width × width).

### Flow cytometry

After treated with different concentration MIL for 48 h, cells were stained with Annexin V-FITC and propidium iodide (PI). Apoptotic cells were measured on a flow cytometer (CytoFlex, Beckman Coulter, USA).

### Caspases activity assays

To measure caspases activity, Caspases-like protease activity in SK-OV-3 and A2780S cells was analyzed using a colorimetric test system. After treating with 4 μM MIL for 36 h, cells were lysed with lysis buffer (Biyotime, China) on ice. Cytosolic protein was mixed with 10 μL caspases-specific substrate (2 mM) for 2 h at 37°C. The value of absorbance of sample was monitored at 405 nm using a Spectramax M5 Microtiter Plate Luminometer.

### Immunofluorescence imaging

Cells were cultured on poly-l-lysine-coated glass coverslips and treated with 4 μM MIL and fixed with 4% formaldehyde polymer for 10 min and subsequently incubated with primary antibodies overnight. The Alexa-fluor-conjugated secondary antibodies were incubated for one hour at room temperature. The nucleus stained with Hoechst 33258. The images were detected using an inverted fluorescence microscopy.

### Western blot

After being treated with various concentrations of MIL, cells were lyed in cell lysis buffer (Biyotime, China) on ice. The protein concentration was determined by Bio-Rad DC protein Assay and 30 μg of each sample was fractionated by SDS-PAGE and transferred to a polyvinylidene difluoride membrane. The primary antibodies incubated overnight. The secondary antibodies incubated 1 h. Proteins were visualized with enhanced chemiluminescence (ECL, Millipore, USA).

### Electromobility shift assay (EMSA)

A specific probe for DNA-protein interaction analysis was used, containing a NF-κB binding element. The following oligonucleotides were used: NF-κB sense, 5-AGCTTCAGAGGGGACTTTCCGAGAGG-3 and NF-κB antisense, 5-TCGACCTCTCGGAAAGTCCCCT CTGA-3. Nuclear extracts and EMSA experiments were performed as the instructions for the Chemiluminescent Nuclei Acid Detection Module (Thermo Fisher Scientific, Waltham, PA).

### SCGE (comet assay)

Cells were treated with or without MIL (4 μM) for 24 hours or 36 hours. The DNA damage of cells was analyzed by SCGE (comet assay) as described by Fairbairn et al. [46]. Cells were embedded in 75 μL 0.5% low-melting point agarose, and microscope slides were immersed in ice-cold lysis buffer (2.5 M NaCl, 100 mM EDTA, 10 nM Tris, 1% sodium laurylsarcosine (pH 10), 1% Triton X-100 and 10% DMSO) for 80 min. Then the slides were exposed to alkali (300 mM NaOH and 1 mM EDTA (pH > 13)) for 40 min. After electrophoresis (25 V, 300 mA, 15 min), the slides were neutralized in 0.4 M Tris buffer (pH 7.5). The ethidium bromide-stained slides were analyzed using image analysis system (Olympus, Tokyo, Japan).

### Topoisomerase II assay

We detected the inhibition function of MIL on topoisomerase II using a topoisomerase II drug screening kit (TopoGEN, USA). Briefly, 1 μL supercoiled DNA was suspended in a reaction buffer. Different concentration of MIL was added to the mixture before the reaction was started by topoisomerase II enzyme addition. The mixture was incubated at 37°C for 30 min, and the reaction was stopped by adding 1 μL 10% SDS. The topoisomerase II enzyme in the mixture was digested by proteinase K (50 μg/mL) at 37°C for 15 min. The DNA samples were cleaned by extracting with chloroform/isoamyl alcohol (24:1, v/v) treatment and analyzed by 1% agarose gel containing 0.5 μg/mL ethidium bromide electrophoresis.

### Molecular docking simulation

In order to investigate the binding interactions between topoisomerase II and MIL (PDB ID: 3QX3) [11], the crystal structure of human topoisomerase II in complex with DNA and etoposide was employed as the docking template. The preparation of complex structure and ligand, molecular docking were accomplished by Discovery Studio 3.1, and the docking results were analyzed by PyMol 1.5. The active site for MIL docking was constituted by the topo II-DNA within 7.5 Å around the etoposide. The best pose was picked out by the docking score and the rationality of molecular conformation.

### Histologic analysis

Tumors and tissues were fixed in 4% neutral buffered formalin solution for more than 24 h and embedded in paraffin. Sections 3–5 μm of the tissues were used to measure the effect of MIL on inducing apoptosis using TUNEL assay (*In Situ* Cell Death Detection Kit, Roche, Mannheim, Germany). The effect of MIL on NF-κB (p65) would be measured using NF-κB (p65) (Mouse, Santa Cruz, sc8008) staining.

### Statistical analysis

All statistical analyses were performed using Student's *t*-test. *P* < 0.05 (*), *P* < 0.01 (**) and *P* < 0.001 (***) were considered as significant (asterisks refer to all Figures).

## SUPPLEMENTARY MATERIALS



## Abbreviations

MIL: Millepachine
DDR: DNA damage response
ATM: ataxia-telangiectasia mutated
ATR: ATM and Rad3-related
DNA-PK: DNA-dependent protein kinase
SSB: DNA single-strand break
DSB: DNA double-strand break
PAC: paclitaxel
CAPE: Caffeic Acid Phenethyl Ester
PI: propidium iodide
EMSA: electromobility shift assay
