# Supplementary material for: Millepachine, a potential topoisomerase II inhibitor induces apoptosis via activation of NF-κB pathway in ovarian cancer
Source: Oncotarget. 2016 Jul 20;7(32):52281–93. doi: 10.18632/oncotarget.10739 (PMC5239551; doi:10.18632/oncotarget.10739)
Supplement: Supplementary file 1 [file oncotarget-07-52281-s001.pdf]

Millepachine, a potential topoisomerase II inhibitor induces apoptosis via activation of NF-κB pathway in ovarian cancer

Supplementary Materials

Supplementary Table 1: MIL inhibited tumor growth in SK-OV-3 and A2780S xenograft models and paclitaxel (PAC)-resistant A2780T xenograft model

Xenograft Models		Tumor Volume (mm^3) (x ± SE)	Tumor Weight (g) (x ± SE)	Inhibitory Rate (% of Control)
SK-OV-3	Con	1179.07 ± 162.76	1.13 ± 0.15	-
	PAC	253.61 ± 31.63	0.24 ± 0.03	78.30
	MIL	198.95 ± 32.83	0.22 ± 0.04	80.31
A2780S	Con	3205.02 ± 354.95	3.44 ± 0.35	-
	PAC	896.26 ± 22.05	0.97 ± 0.28	71.91
	MIL	785.08 ± 102.23	0.83 ± 0.10	75.79
A2780T	Con	3481.94 ± 386.52	3.52 ± 0.30	-
	PAC	2449.23 ± 191.66	2.51 ± 0.25	29.03
	MIL	1106.10 ± 277.71	1.46 ± 0.06	58.48

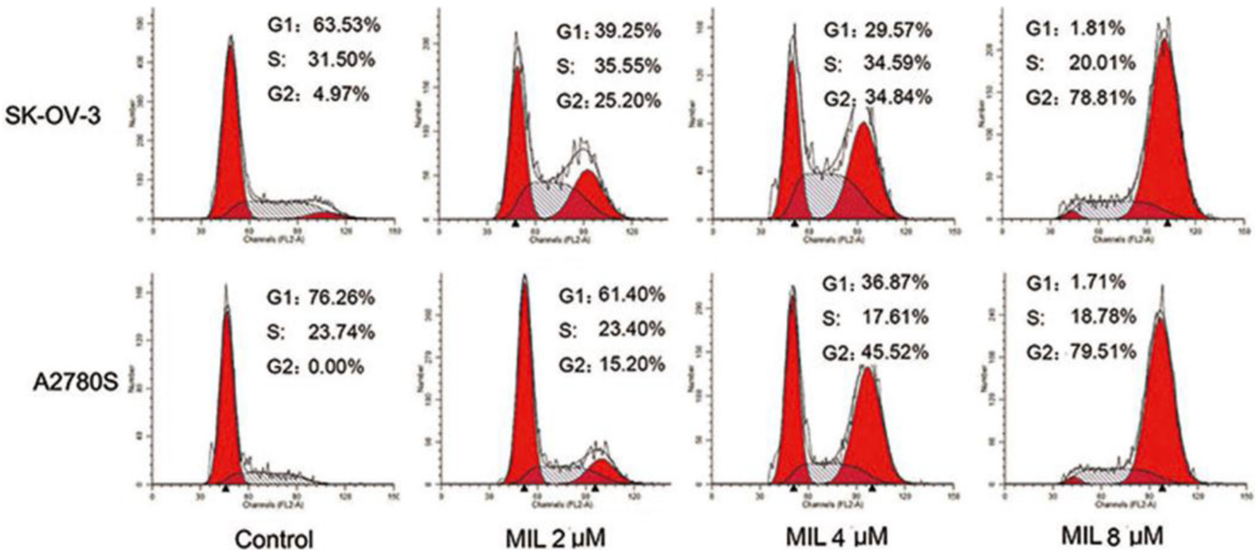

Supplementary Figure S1: MIL induced G2/M arrest in SK-OV-3 and A2780S cells within treating for 24 h. SK-OV-3 and A2780S cells (2 × 10^5) were cultured in 6-well cell culture plates and treated with different concentrations of MIL for 24 h. After treatment, cells were stained with PI and analyzed by a flow cytometer (TASC240, USA). Data were analyzed using Modfit 2.8 software.

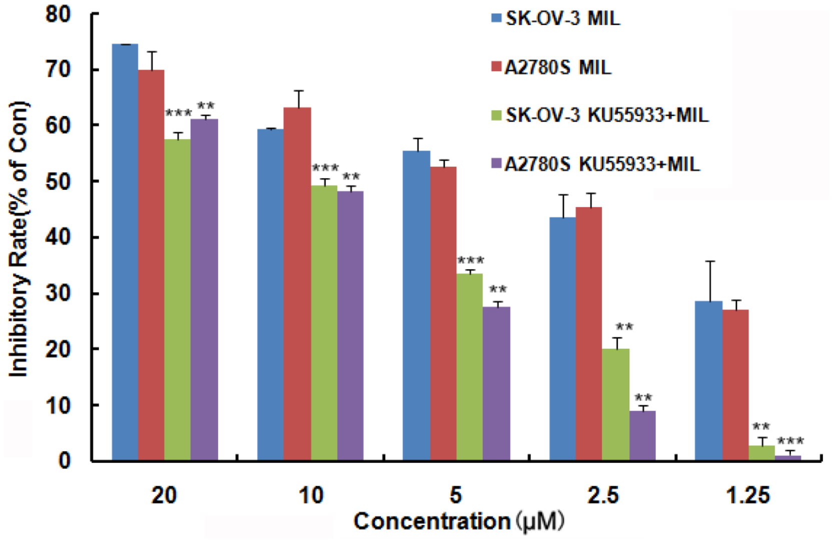

Supplementary Figure S2: ATM inhibitor alleviated effect of MIL on the proliferation of SK-OV-3 and A2780S cells. SK-OV-3 and A2780S cells were pre-treated with 10 μM KU55933 (a specific inhibitor of ATM) for 2 h, then treated with different concentrations of MIL combined with 10 μM KU55933 for 48 h. After treatment, to detect the proliferation inhibition of MIL suing MTT assay.

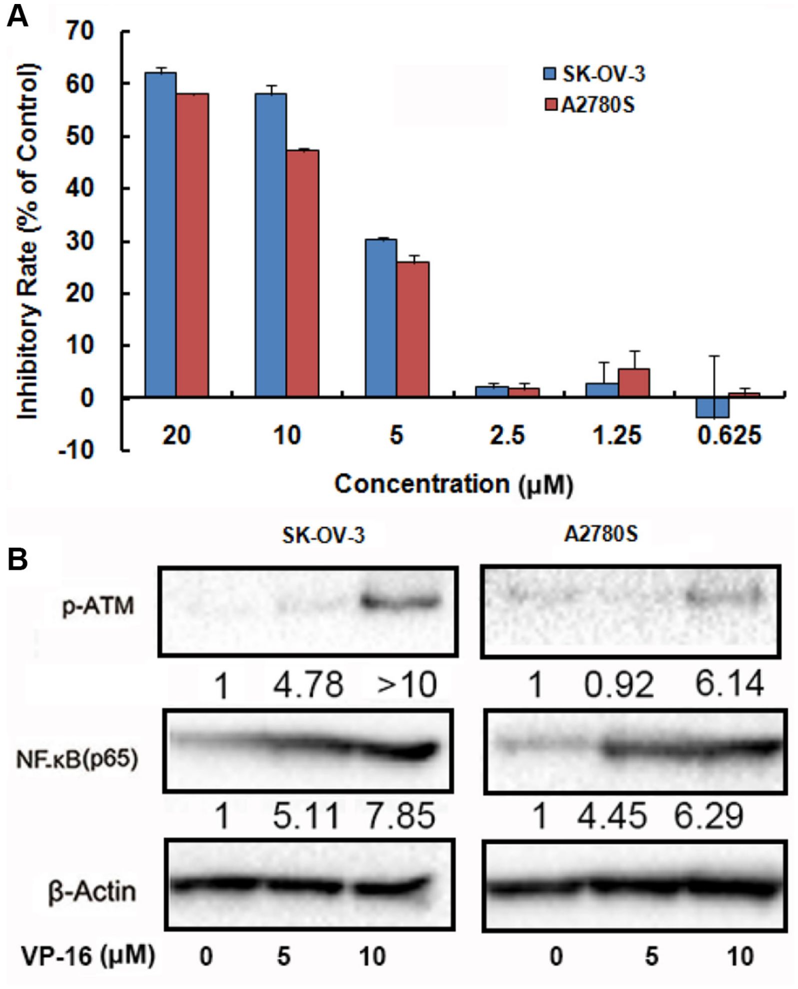

Supplementary Figure S3: VP-16 inhibited the proliferation (A) and increased the expression of p-ATM and NF-κB (p65) (B) in SK-OV-3 and A2780S cells. (A) SK-OV-3 and A2780S cells (2×10^5) were cultured in 6-well cell culture plates and treated with different concentrations of VP-16 for 48 h. After treatment, to detect the proliferation inhibition of MIL suing MTT assay. (B) SK-OV-3 and A2780S cells were treated with 5 μM and 10 μM of VP-16 for 48 h. The protein levels of p-ATM and NF-κB (p65) were determined using western blot assay.
